# Citric Acid and Sodium Bicarbonate as an Alternative Carbon Dioxide Source for Mosquito Surveillance

**DOI:** 10.3390/insects16010090

**Published:** 2025-01-16

**Authors:** Christine Hong, Victoria J. Brookes, Ruth N. Zadoks, Cameron E. Webb

**Affiliations:** 1 Sydney School of Veterinary Science, Faculty of Science, The University of Sydney, Camperdown, NSW 2006, Australiavictoria.brookes@sydney.edu.au (V.J.B.); ruth.zadoks@sydney.edu.au (R.N.Z.); 2 Sydney Infectious Diseases Institute, Faculty of Medicine and Health, The University of Sydney, Camperdown, NSW 2006, Australia; 3 School of Medical Sciences, Faculty of Medicine and Health, The University of Sydney, Camperdown, NSW 2006, Australia; 4 Department of Medical Entomology, NSW Health Pathology, Institute of Clinical Pathology and Medical Research, Westmead Hospital, Westmead, NSW 2145, Australia

**Keywords:** mosquito surveillance, citric acid, sodium bicarbonate, dry ice, carbon dioxide production

## Abstract

Most mosquito surveillance programs rely on traps baited with carbon dioxide (CO_2_) to attract host-seeking mosquitoes. The source of CO_2_, traditionally dry ice or gas cylinders, poses operational challenges, especially in remote locations. CO_2_ production from citric acid and sodium bicarbonate (NaHCO_3_) using low-cost intravenous fluid bags (‘acid traps’) was evaluated in laboratory experiments. Field trials then compared the efficacy of these acid traps with dry ice traps. Using a 2 × 2 Latin square style design, trapping locations were established at two sites in Sydney, New South Wales, Australia (one urban and another peri-urban), to assess trap performance in diverse environments. The laboratory results showed CO_2_ production of sufficient amount and duration for overnight mosquito capture. Whilst field experiments showed that dry ice traps captured more mosquitoes (up to 59%) than acid traps, numbers were similar in the urban environment. At both sites, species composition was similar, with *Culex quinquefasciatus*, *Aedes notoscriptus*, and *Aedes vigilax* predominantly collected, and these are known species of pest and public health concern. Further modifications to the acid traps could further improve CO_2_ output and trap efficiency.

## Simple Summary

Trapping mosquitoes that are known vectors of pathogens of human health concern is essential for public health surveillance. Commonly deployed mosquito traps typically include carbon dioxide (CO_2_) supplied via compressed gas or dry ice, which can be expensive and difficult to transport. This study explored an alternative CO_2_ source for attracting mosquitoes using citric acid and baking soda (sodium bicarbonate) in a novel CO_2_ delivery system. Laboratory experiments assessed the effectiveness of CO_2_ production using citric acid and baking soda, followed by field trials in two locations to compare capture rates to dry ice traps. The findings showed that citric acid and baking soda could be an alternative to traditional CO_2_ sources in mosquito traps.

## 1. Introduction

Mosquito-borne diseases cause a significant public health burden worldwide, infecting hundreds of millions of people annually, with an increased incidence within the last two decades [1]. With over 75 different arboviruses identified in Australia, including pathogens such as Dengue (DENV), Japanese encephalitis (JEV), Murray Valley encephalitis (MVEV), and Ross River (RRV) viruses, it is important to monitor mosquito populations to assist health authorities in making decisions on public health risk management [2,3]. These viruses are responsible for causing a range of illnesses, from mild symptoms to severe neurological complications, presenting challenges to healthcare systems and community well-being [4]. Moreover, zoonotic mosquito-borne viruses (ZMBVs) pose significant implications for animal health [5], with dog heartworm (*Dirofilaria immitis*) a concern for pet owners and JEV affecting the health of pigs [6,7]. The interconnection between human and animal health underscores the importance of understanding these vectors and their role in transmitting zoonotic diseases [5].

Without broadscale mosquito control programs, health authorities in Australia primarily rely on raising awareness of elevated mosquito-borne disease risk to minimise public health threats. Effective mosquito surveillance is a tool for early detection and response to elevated or emergent virus activity, allowing authorities to implement timely strategic responses [8]. In Australia, mosquito surveillance programs, such as the use of sentinel animals and mosquito-based trapping methods, have been important in detecting and monitoring arbovirus activity [9] Mosquito surveillance can provide valuable insights into disease transmission dynamics, facilitating the prediction of virus fluctuations and the establishment of baseline activity levels [3,8].

Mosquito traps, such as the Encephalitis Virus Surveillance (EVS), Bioagents BG-Sentinel (BGS), and Centres for Disease Control and Prevention (CDC) traps, are essential tools for mosquito surveillance [10]. A variety of attractants are employed with these traps to enhance effectiveness [10]. Light is used with CDC and EVS traps, but when used alone, it is deemed insufficient and may lead to unwanted bycatch and mosquito specimen damage [10] For attracting host-seeking anthropophilic mosquito species, artificial human skin scents such as BG-Lure (BGL) or BG-Sweetscent (BGSw) are recommended for use with BGS and BGM traps [11]. Most commonly, health authorities supplement traps with CO_2_ to significantly increase both the quantity and diversity of captured mosquitoes [12] and the two main CO_2_ sources used are dry ice and compressed gas [11]. Both methods have logistical constraints, safety concerns, or high costs associated with implementation [13,14]. Dry ice is affordable and more widely used than compressed gas, but its effectiveness can vary due to variability in release rates under different environmental conditions, and it is difficult to source dry ice outside major metropolitan areas [11,15,16].

Citric acid combined with sodium bicarbonate may offer a cost-effective and easily transportable alternative for CO_2_ generation in remote locations where the use of gas cylinders or dry ice is impractical [17]. This method uses readily available, non-hazardous materials that can be bulk shipped without regulatory restrictions, making it a viable option for mosquito surveillance [18]. However, while this approach shows promise, potential limitations have been highlighted in maintaining consistent CO_2_ production levels throughout the night. Citric acid and sodium bicarbonate traps can initially generate substantial amounts of CO_2_, but the output often declines over time. One device using crushed coquina shells and citric acid produced a high initial output of approximately 550 mL/min but dropped to around 150 mL/min after four hours, leading to a 70% reduction in mosquito collection compared to dry ice traps [18]. Similarly, another device delivered 250 mL/min of CO_2_ initially, declining to 50–60 mL/min after 12 h [12]. These findings suggest that while acid-carbonate systems provide a practical alternative, they may require further refinement to match the performance of dry ice, particularly in sustaining CO_2_ output for extended periods.

The objective of this study was to evaluate and compare traps using citric acid and NaHCO_3_-based CO_2_ production method in a novel generator device (referred to as ‘acid trap’) with commonly used dry ice-based CO_2_ production methods (referred to as ‘dry ice trap’) considering both the mosquito numbers and mosquito species that are trapped. The study’s outcomes were intended to assist authorities in New South Wales, Australia, to enhance surveillance strategies for mosquito-borne disease management in rural areas in which dry ice or CO_2_ cylinders are not available or operationally difficult to deploy.

## 2. Materials and Methods

The study was conducted in two phases. Phase 1 involved a laboratory investigation to assess CO_2_ generation from citric acid and NaHCO_3_ under controlled conditions. This preliminary phase aimed to establish the optimal concentration to be used with traps. Phase 2 was a field trial comparing mosquito capture between acid and dry ice traps.

### 2.1. Generator Device

**Figure 1:**
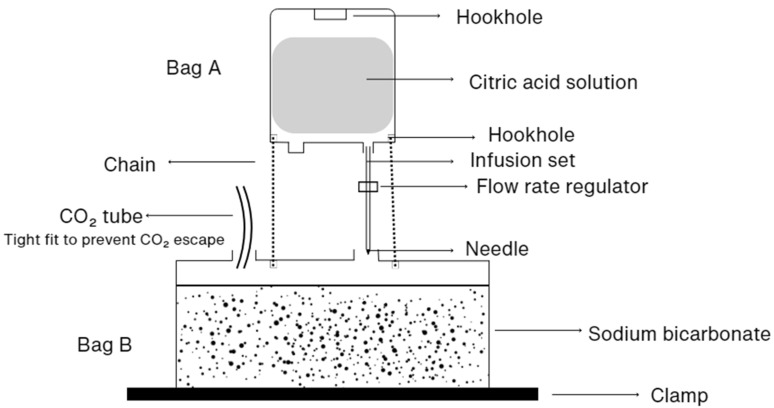
Modification of two intravenous drip bags for use as CO_2_ generators in mosquito traps.

The generator device (Figure 1) used in this study consisted of two modified intravenous (IV) drip bags. Bag A (1 L) was filled with citric acid solution (Blants Citric Acid, USP & Food Grade, ≥99% purity; Blants Wellbeing & Lifestyle, Sydney, Australia), and Bag B (5 L) with NaHCO_3_ powder (Blants Natural Sodium Bicarbonate, USP & Food Grade, Aluminium-free, 100% purity; Blants Wellbeing & Lifestyle, Sydney, Australia). The top corner of Bag A was cut to allow filling, and Bag B was suspended 20 cm below Bag A. The upper portion of Bag B was opened to add NaHCO_3_, then securely sealed with clamps. To minimise empty space and ensure even distribution of the citric acid into Bag B, the excess bag material was rolled and clamped.

A standard IV giving set connected Bag A to Bag B using an “18Gx1.5” Agani Hypodermic Needle (Terumo Medical Corporation, Macquarie Park, Australia), with a flow rate controller regulating liquid from Bag A at 1.5 mL/min for a 10 h period (30 drops/min). Tubing cut from another IV set connected to Bag B allowed CO_2_ to escape, with 1 m of tubing leading to the mosquito trap. In the field study, excess tubing was secured to the chains with masking tape to prevent movement during adverse weather. The CO_2_ tubing was inserted deep into Bag B’s injection port to ensure CO_2_ drainage and reduce the risk that Bag B bursts.

Laboratory trials were conducted at an estimated temperature of 18–22 °C to determine optimal CO_2_ concentration and flow rate. The citric acid concentration was initially set at 300 g in 1000 mL of water and later increased to 500 g in 550 mL. Gas production was measured using a water displacement method. The final configuration was repeated six times to verify consistency in CO_2_ production.

### 2.2. Field Investigation Study Sites

**Figure 2:**
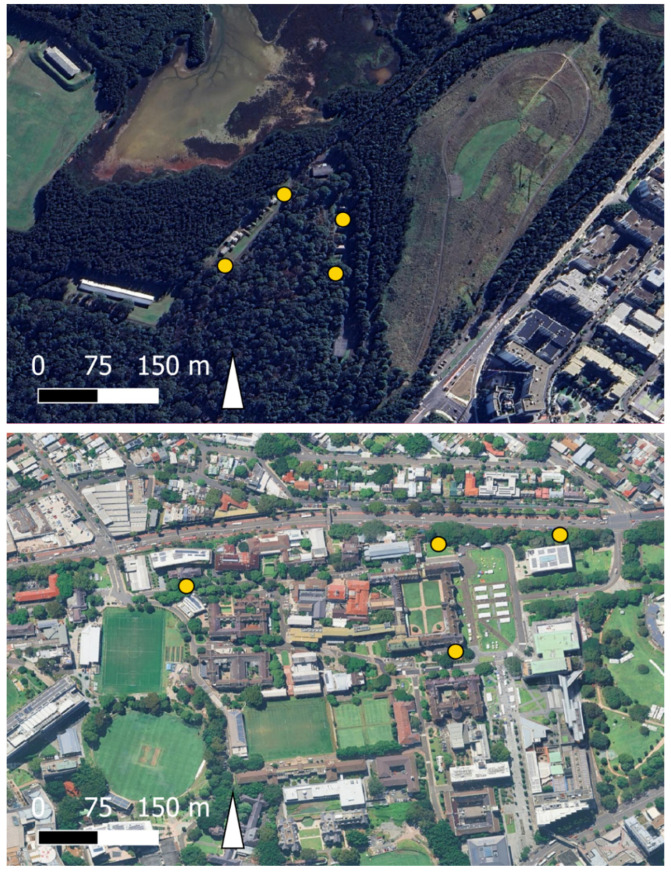
Satellite images of the study locations demonstrating the different landscape characteristics. Newington Nature Reserve (**top**), 33.8281° S, 151.0611° E, and University of Sydney (**bottom**), Camperdown, 33.8884° S, 151.1868° E. Yellow dots indicate trap sites where paired trap types were tested. Map produced by authors using QGIS version 3.38.3-Grenoble (http://qgis.org; accessed on 5 December 2024).

The field trial involved two treatment types (either citric acid and NaHCO_3_ or dry ice as the source of CO_2_). Two locations were selected for testing to represent different landscape types and concomitant mosquito communities. Adult mosquito collection took place from 12th February to 14th February 2024, at the University of Sydney (USYD), Camperdown (33.8884° S, 151.1868° E), and 21st February to 23rd February at Newington Nature Reserve (NNR), Sydney Olympic Park (33.8281° S, 151.0611° E). These locations represented two distinct environments: an urban landscape at USYD (Figure 2), characterised by open lawns and wooded spaces amongst buildings, and a woodland area at NNR (Figure 2) featuring dense forest and adjacent freshwater and estuarine wetland habitats. Traps at both sites were placed at least 50 m apart to minimise interference and ensure consistent data collection, allowing for repeatability in future experiments.

With an expected large effect on mosquito catch numbers between trap types (d = 0.8), a significance level of α = 0.05, and a power of 0.8 (1 − β), 10 traps per trap type were estimated to be required. This was not feasible due to resource constraints but a compromise of eight traps per trap type was considered appropriate. At each of the two locations, a total of four trap sites were established, with each site having a pair of trap types (i.e., one with CO_2_ provided by dry ice and another provided by the citric acid and NaHCO_3_) operated approximately 20 m apart over two consecutive nights. Trap types were rotated between nights according to a 2 × 2 Latin square design to ensure that each of the trap types was operated at each of the individual trap sites to prevent potential biases due to specific individual trap sites.

#### Field Investigations Mosquito Trap Comparisons

**Figure 3:**
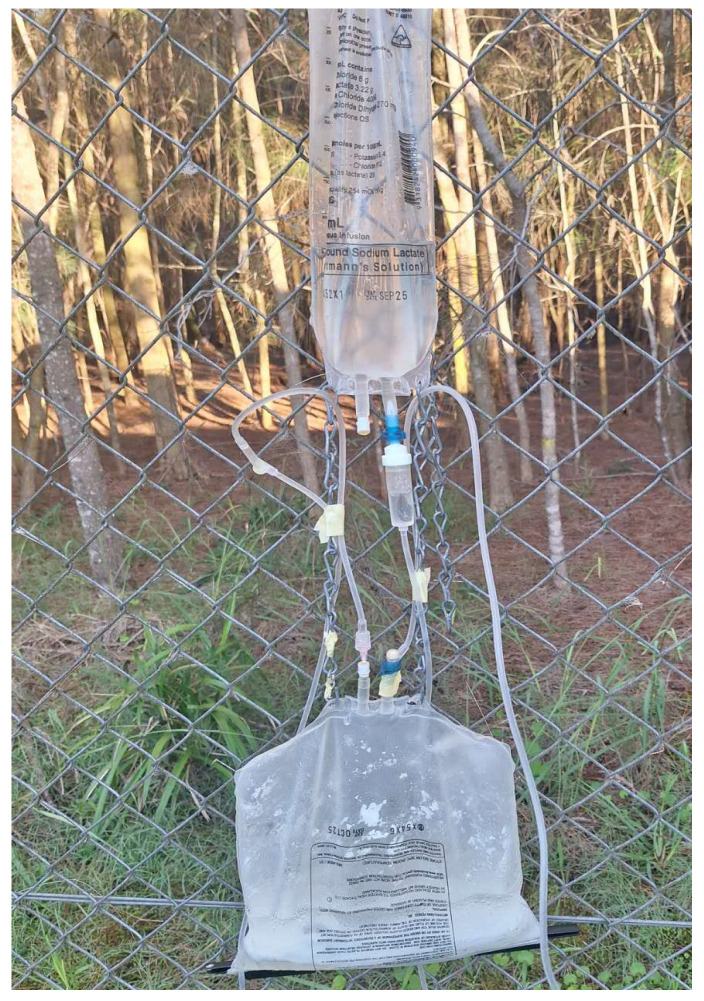
An example of the citric acid generator device used in conjunction with an EVS trap (not shown) for field testing in the Newington Nature Reserve, Sydney Olympic Park, NSW Australia.

Dry ice traps (4/test night) used approximately 1.5 kg of dry ice per trap to give an estimated CO_2_ flow rate of 300 mL/min. Each of the acid traps used 500 g of citric acid and 500 g of NaHCO_3_ in the generator device (Figure 3), to give an expected CO_2_ flow rate of 150 mL/min. The operational cost of dry ice per night can be up to $18, while citric acid and NaHCO_3_ were sourced from a commercial food supplier, with operational costs estimated at $3.10 for citric acid and $2.70 for NaHCO_3_ per night (See Figure S1 for operational costs details).

Adult mosquito collections were made using EVS-style traps that include a battery-powered incandescent light and motorised fan to draw mosquitoes into a plastic catch bucket [19]. Traps were baited with a source of CO_2_ provided by either dry ice or the citric acid and NaHCO_3_ device. For the two study locations, all trap configurations at all sites were operated at approximately 1 m above the ground. Temperature and humidity were recorded on days of trapping using 3 p.m. weather observations from the Bureau of Meteorology [20].

Traps were activated between 4 and 6 p.m., and catch buckets were collected between 7 and 8 a.m. the following morning. Drip bags were emptied and rinsed with tap water before being dried for subsequent use. Mosquitoes in the catch buckets were frozen at −20 °C for 20 min to immobilise mosquitoes before transferring them to labelled petri dishes. The specimens were then stored in freezers at −20 °C until they were counted and identified to species level using a pictorial guide and taxonomic keys [21].

The data collected from the laboratory tests were recorded in Excel spreadsheets. Mosquito count data and species were compared between treatments within (but not between) trap sites (USYD, NNR) and visualised using bar plots (generated using R 4.3.3 Feb 2024; R Core Team (2022)). A table summarised the number of species collected in each trap. Additionally, the average Shannon Diversity Index was calculated for each treatment at both sites. The Shapiro test for normality and the Levene test for homogeneity of variances were conducted to assess for non-normal and non-homoscedastic mosquito count data. Statistically significant differences in mosquito counts between trap types were assessed using the Mann–Whitney (MW) U test (α = 0.05) for all mosquitoes combined, as in similar studies [22,23,24,25], and for species with a total count above 50.

## 3. Results

### 3.1. Carbon Dioxide Production

**Figure 4:**
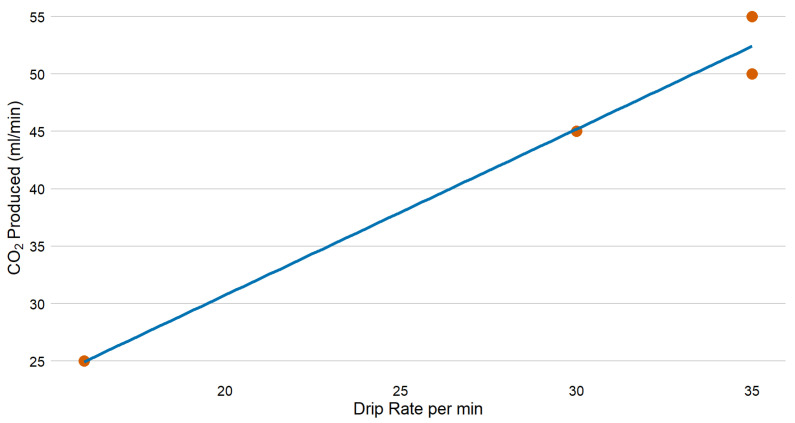
CO_2_ starting flow rates for optimisation tests were generated by the reaction of a citric acid solution (300 mL dissolved in 1 L of tap water) dripped onto 200 g of NaHCO_3_.

In laboratory optimisation tests, 300 g of citric acid dissolved in 1 L water yielded a CO_2_ production rate of up to 40–50 mL/min (Figure 4). To achieve the desired CO_2_ production rate of 150 mL/min, the citric acid concentration would need to be increased threefold to 900 g/L, assuming a linear relationship, but due to the limited size of the generator device (i.e., 1 L), a compromise was required of 500 g citric acid dissolved in 550 mL of water. With a drip rate of 30 drips/min, this configuration provided a citric acid solution flow of 1.5 mL/min and sustained CO_2_ production for a duration of 10 h (Table 1).

**Figure 5:**
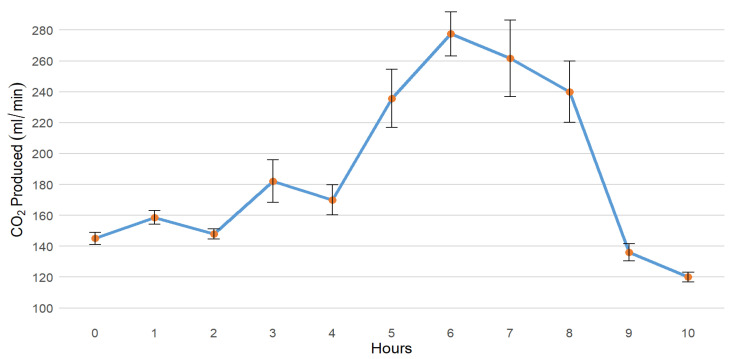
Mean and standard error of CO_2_ flow rates for calibration tests (trial 9 to 15) using citric acid solution (500 g citric acid dissolved in 550 mL tap water) combined with 500 g NaHCO_3_, with a flow rate of 30 drips per minute.

Once the optimised citric acid concentration and amount were established, CO_2_ calibration tests conducted over 10-h periods revealed variation in CO_2_ production (Figure 5). Trials 9 and 10 achieved the target flow rate of 150 mL/min from the initiation of trials. Trials 11, 13, 14, and 15 began with a flow rate of 140 mL/min, while trial 12 started with 135 mL/min. In all trials, CO_2_ production initially increased, reaching a peak before gradually stabilizing. An abrupt decline was observed at approximately 8 h, followed by a stable output of approximately 100 mL/min between 9 and 10 h. This suggests that, despite the initial fluctuations, the CO_2_ generator device can produce a flow rate sufficient for extended trapping, potentially lasting for at least 12 h. Trial 15 with a 5 L drip bag for NaHCO_3_ demonstrated a consistent increase in CO_2_ production, with peak production at 8 h. The highest CO_2_ flow rate observed was 360 mL/min during the 6th hour (Trial 13), while the lowest was 110 mL/min at the 10th hour (Trials 13).

### 3.2. Field Investigations Mosquito Collections

No rainfall was recorded during either of the field trials. Temperatures ranged from 25.9 °C to 30.2 °C at the USYD and NNR sites, respectively. A total of 1003 and 4730 mosquitoes were captured from USYD and NNR, respectively (Tables S1 and S2). The dry ice and acid traps at USYD collected a median of 273 mosquitoes/night (95% range: 158.05–387.95) and 228.5 mosquitoes/night (95% range: 224.23–232.78), respectively (Mann–Whitney U [MWU] test, U = 31, *p* = 0.9). At NNR, the dry ice traps collected a median of 1556.5 mosquitoes/night (95% range: 1152.28–1960.73), and the acid traps collected 915 mosquitoes/night (95% range: 604.35–1225.65) (MWU test, U = 8.5, *p* = 0.01). Most of the species counts showed a substantial difference between dry ice-based traps and acid traps (Figures S2 and S3).

At USYD, traps collected four species with the two most abundant species (*Aedes notoscriptus* and *Culex quinquefasciatus*) comprising 99.3% of the total catch (Table S1). Newington Nature Reserve exhibited greater species richness, with 17 species identified (Table 2). The seven most abundant species (*Ae. alternans*, *Ae. notoscriptus*, *Ae. vigilax*, *Anopheles annulipes*, *Coquillettidia linealis*, *Cx. annulirostris*, and *Cx. sitiens*) accounted for 98.28% of the total catch (Table 2).

For species in USYD, the number of *Cx. quinquefasciatus* showed significant difference between the two trap types (*p* = 0.01), while a significant difference between trap types was observed for the number of mosquitoes within three species in NNR; *Ae. vigilax* (*p* = 0.01), *An. annulipes* (*p* = 0.0009) and *Cx. annulirostris* (*p* = 0.01) (Table 2).

A comparison of mosquito catches between the two treatments demonstrated overall similar collection patterns across most locations. At NNR, dry ice traps consistently outperformed citric acid traps, evidenced by both higher mosquito counts and average Shannon Diversity Index (SDI). This indicates greater species diversity with the dry ice traps at this site. In contrast, at the USYD, five out of eight locations showed marginally higher mosquito catches with citric acid traps; however, the overall mosquito collection at these locations was low. Notably, the SDI for citric acid traps (0.516) surpassed that of dry ice traps (0.397), suggesting a slightly broader range of species captured in this urban environment (Table 3). While significant differences in catches were observed at certain sites (Figures S4 and S5), the dry ice trap results from USYD, Site2 2, may represent an outlier (Figure S4).

Observations during the trials revealed some practical issues with the citric acid traps. The generator’s chains showed visible damage (i.e., possible corrosion) after four days of use, and incomplete reactions of NaHCO_3_ occasionally caused bag inflation by morning.

## 4. Discussion

This study demonstrates that where mosquitoes of pest and public health importance are present, they can be collected by traps using either dry ice or the combination of citric acid and NaHCO_3_ as CO_2_ sources. The abundant species collected in this project are known for their potential to cause nuisance bites and transmit pathogens to humans in Australia [21]. The results also demonstrated that whilst dry ice might be a more effective source of CO_2_ for collecting high numbers of mosquitoes when compared with citric acid and NaHCO_3_ in some settings, the performance of the citric acid and NaHCO_3_ as a CO_2_ source for mosquito traps is comparable in some circumstances and could be sufficient for mosquito surveillance purposes. The lack of a significant difference between the two trap types at USYD, where overall mosquito abundance was low, suggests that in circumstances such as these, traps baited with CO_2_ from either source may be equally effective.

There are a number of potential reasons for the significant difference observed between the treatments at NNR. It might be due to low or unsteady flow rates of citric acid onto NaHCO_3_ because this site was more exposed to wind than USYD and tubing might have been disturbed, or the predominant mosquito species at NNR might need overall higher CO_2_ flow rates for attraction. The relationship between CO_2_ flow rates and mosquito capture rates demonstrates varying affinities among different mosquito species [14]. Studies have shown that for species such as *Ae. albopictus*, collections increased significantly with rising CO_2_ levels, peaking at 600 mL/min [14]. While female collections plateaued beyond 300 mL/min, males continued to increase with higher flows [15]. Although absolute mosquito numbers trapped with the citric acid traps were lower than with dry ice traps, the species composition was barely affected.

The CO_2_ generator designed for this study has a capacity of 1.2 L for the citric acid solution and can hold 500 g of NaHCO_3_. Maintaining a consistent flow rate of around 30 drips per minute was crucial for this experiment. However, after several trials, it became evident that the CO_2_ flow rate gradually decreased after an initial peak. It remains unclear whether this was due to decreased citric acid solution drip rates as Bag A emptied and became lighter or if there were changes in the contact rate between citric acid and NaHCO_3_ as the NaHCO_3_ in Bag B was gradually consumed. Carbon dioxide generated from citric acid solution typically produces 150 mL/min at the start of the reaction increasing to about 300 mL/min at 6 h and decreasing to 100 mL/min at 10 h (Figure 5). The flow rate could be higher than 30 drips/minute to compensate for reduced CO_2_ production later. Although CO_2_ production is not at the same level throughout the night, gas production typically reaches a steady state between 6 and 9 h after starting, maximising output during the period when *Aedes* mosquitoes are most active [26] but less with the dawn and dusk feeding peak observed in many *Culex* mosquitoes [27]. However, the results of our investigation did not suggest a strong bias in the collection of either group of mosquitoes. Constructed primarily from recycled IV drip bags, chains, and clamps, this cost-effective device delivers reasonable performance with minimal maintenance requirements. However, based on observations during the trials, the device’s lifespan may be short, as the chains began to show signs of damage after just four days, indicating a need for regular replacement or the consideration of alternative materials, such as plastic chains or strings. Occasionally, incomplete chemical reactions of NaHCO_3_ may cause excessive bag inflation that may result in damage and require more frequent replacement. Long-term field trials are necessary to assess the generator’s durability under various environmental conditions, including variable conditions of temperature and humidity. However, it should be noted that mosquito surveillance is rarely undertaken under consistent environmental conditions and there needs to be consideration given to the operational constraints when expectations of specific CO_2_ generation rates are specified. To ensure a consistent flow rate throughout the trapping period, future experiments could explore the use of IV flow regulators. However, it is essential to note that multiple studies have reported significant variation in flow regulator performance [28]. Therefore, it is recommended to conduct laboratory studies with flow regulators before implementing them in field studies.

Potential challenges to applying this approach to mosquito surveillance also include the costs associated with reagents and the requirement for a water supply to prepare acidic solutions in rural areas [18]. Transporting water to the trapping location adds considerable weight to the trapping supplies [18]. Alternatively, natural water sources near trapping sites could be used, though preliminary studies to assess the impact of water sources are necessary in such cases [18]. During the development of the generator device, considerations were made regarding the use of effervescent tablets as potential CO_2_ sources. These tablets typically contain a mixture of citric acid and NaHCO_3_, releasing carbon dioxide gas when dissolved in water [29]. While they may produce some CO_2_, the quantities generated may not be sufficient for certain applications, such as mosquito trapping [29]. However, exploring the use of effervescent tablets could provide insights into potentially simpler methods for CO_2_ production using the same chemicals as the current study.

The limitations of this study were the limited number of experimental sites and replication, as well as the use of dry ice instead of cylinders for the comparison to citric acid and NaHCO_3_. Future research on this approach should initially assess the comparability of results obtained using gas cylinders, because they can provide CO_2_ in controlled flow rates at any time [30]. Additional work should also aim to determine the optimal trapping session duration, examining both overnight sessions (exceeding 12 h) and shorter intervals (less than 4 h). This is especially the case for mosquito species of public health interest, which might exhibit a differing response to release rates of CO_2_ or have circadian activity and host-seeking activity that does not align with periods of sufficient CO_2_ production [31]. While the overnight field experiment in this study aimed to maximise mosquito diversity, shorter trapping periods might be suitable for targeted surveillance, given the varying activity patterns of mosquito species [32] and increased capture of highly mammalophilic mosquitoes with higher CO_2_ concentrations [33]. By increasing the CO_2_ concentration within shorter time frames, it would be possible to target specific mosquito species. For instance, prioritising the monitoring of specific arbovirus vectors may be more important than focusing on other species, such as the investigation into vector activity during the recent JEV outbreak in southeast Australia [34]. Therefore, optimising trapping methods to effectively capture these specific vectors is crucial for disease surveillance and control. This approach supports the potential benefits of using citric acid-based methods, especially in remote regions like the Northern Peninsula Area of Queensland, in which JEV has previously been detected and is geographically close to high-risk regions for JEV transmission [35]. Similarly, mosquito trapping in remote regions within the Murray Darling Basin, where JEV and MVEV surveillance is considered important, but logistically difficult, may be assisted by these alternative sources of CO_2_. Mosquito research in remote rural locations may also benefit from this approach when reliant on CO_2_-baited trapping technologies for specimen collection. In such areas, where access to resources like gas cylinders may be limited, acid traps could provide a practical and effective solution for targeted surveillance of medically significant mosquito species.

## 5. Conclusions

The development and testing of mosquito traps baited with CO_2_ generated by citric acid and NaHCO_3_ presented in this study demonstrate that mosquitoes of pest and public health importance are readily collected. This study provides promising implications for enhancing Australia’s mosquito surveillance system, particularly in remote and rural areas. Traditional surveillance methods that rely on resources like dry ice or compressed CO_2_ encounter logistical and cost challenges in these regions. This study suggests that acid traps could be worth evaluating to see if they can improve mosquito surveys in these locations as they rely on easily accessible materials and a straightforward design. Given the significant public health concerns associated with mosquito-borne diseases in Australia, the trap’s efficacy in capturing species related to arboviruses highlights its potential use in monitoring mosquito populations.

## Figures and Tables

**Table 1:** Experimental trials tested citric acid concentration, sodium bicarbonate amount, and flow rates for CO_2_ production.

Trials	Citric Acid Solution	Sodium Bicarbonate (g)	Flow Rate (Drops/min)	Amount of Citric Acid Solution (mL/min)	CO_2_ Production Rate (mL/min)—Starting Amount
Trial 1	300 g/1000 mL	200	16	0.8	25
Trial 2	300 g/1000 mL	200	30	1.5	45
Trial 3	300 g/1000 mL	200	35	1.8	55
Trial 4	300 g/1000 mL	200	35	1.8	50
Trial 5	500 g/550 mL	500	38	1.9	180
Trial 6	500 g/550 mL	500	48	2.4	210
Trial 7	500 g/550 mL	500	14	0.7	50
Trial 8	500 g/550 mL	500	34	1.7	180
Trial 9	500 g/550 mL	500	30	1.5	150
Trial 10	500 g/550 mL	500	30	1.5	170
Trial 11	500 g/550 mL	500	30	1.5	140
Trial 12	500 g/550 mL	500	30	1.5	135
Trial 13	500 g/550 mL	500	30	1.5	140
Trial 14	500 g/550 mL	500	30	1.5	140
Trial 15	500 g/550 mL	500	30	1.5	140

**Table 2:** Mean and standard error (SE) of mosquito species counts in citric acid and dry ice traps, percentage distribution of species count, and Mann–Whitney U (MWU) test results for differences in mosquito counts by species. Mosquitoes were collected from traps at the University of Sydney and Newington Nature Reserve.

Species	Dry Ice (Mean, SE)	Citric Acid (Mean, SE)	Percentage in Dry Ice and Citric Acid Treatment (%/%)	*p*-Value (MWU)	Total
University of Sydney					
*Aedes notoscriptus*	50.6 ± 26.0	28.2 ± 7.8	74.2/49.5	0.9	631
*Culex annulirostris*	0.1 ± 0.1	0.0 ± 0.0	0.2/0.0	NA	1
*Culex molestus*	0.1 ± 0.1	0.6 ± 0.3	0.2/1.1	NA	6
*Culex quinquefasciatus*	17.4 ± 7.2	28.2 ± 12.1	25.5/49.5	0.02	365
Newington Nature Reserve					
*Aedes aculeatus*	0.6 ± 0.3	0.8 ± 0.4	0.2/0.4	NA	11
*Aedes alternans*	15.5 ± 3.8	8.1 ± 2.0	3.9/3.7	0.2	189
*Aedes multiplex*	0.1 ± 0.1	0.1 ± 0.1	0.0/0.1	NA	2
*Aedes notoscriptus*	10.1 ± 3.8	9.9 ± 3.6	2.6/4.4	0.6	160
*Aedes procax*	2.5 ± 0.5	0.8 ± 0.4	0.6/0.4	NA	11
*Aedes vigilax*	254.1 ± 44.1	128.8 ± 20.8	65.3/57.9	0.01	3063
*Anopheles annulipes*	23.1 ± 7.2	2.8 ± 0.7	5.9/1.2	0.0009	207
*Coquillettidia linealis*	22.2 ± 6.7	11.4 ± 2.0	5.7/5.1	0.2	269
*Coquillettidia xanthogaster*	0.3 ± 0.2	0.3 ± 0.2	0.1/0.1	NA	4
*Culex annulirostris*	36.0 ± 5.0	25.0 ± 5.2	9.3/11.3	0.01	488
*Culex molestus*	0.6 ± 0.3	0.0 ± 0.0	0.2/0.0	NA	5
*Culex orbostiensis*	0.8 ± 0.4	0.3 ± 0.2	0.2/0.1	NA	8
*Culex quinquefasciatus*	0.9 ± 0.5	0.0 ± 0.0	0.2/0.0	NA	7
*Culex sitiens*	21.4 ± 5.8	12.8 ± 1.6	5.5/5.7	0.1	273
*Mansonia uniformis*	0.8 ± 0.5	0.3 ± 0.2	0.2/0.1	NA	8
*Tripteroides atripes*	0.1 ± 0.1	0.0 ± 0.0	0.0/0.0	NA	1
*Verrallina funerea*	0.0 ± 0.0	0.1 ± 0.1	0.0/0.1	NA	1

NA: Species with collections of fewer than 50 specimens were excluded from statistical comparisons.

**Table 3:** Average Shannon Diversity Indices (SDI) for trapped mosquito species and standard error (SE) at four stations across two sites.

Sites	Dry Ice (SDI, ME)	Citric Acid (SDI, ME)
University of Sydney	0.397 ± 0.086	0.516 ± 0.095
Newington Nature Reserve	2.522 ± 0.036	2.103 ± 0.040

## Data Availability

The datasets of the current study are available in Supplementary Tables S1 and S2 and all other data are available on request from the corresponding author.

## Supplementary Materials

The following supporting information can be downloaded at: https://www.mdpi.com/article/10.3390/insects16010090/s1. Table S1: Mosquito trapping data for University of Sydney, Camperdown, by location, date and trapping method. Ae = *Aedes*, Cx = *Culex*; Table S2: Mosquito trapping data for Newington Nature Reserve, Sydney Olympic Park, by location, date and trapping method. Ae = *Aedes*, An = *Anopheles*, Cq = *Coquillettidia*, Cx = *Culex*, Ma = *Mansonia*, Tp = *Tripteroides*, Ve = *Verrallina*; Table S3: CO_2_ cost per night per source, February 2024. Operational cost (1 night) excludes cargo change; Figure S1: Distribution of mosquito species captured at the University of Sydney, Camperdown, using citric acid-based (CA) and dry ice-based (DI) traps; Figure S2: Distribution of mosquito species captured in Newington Nature Reserve using citric acid-based (CA) and dry ice (DI)-based traps; Figure S3: Comparison of mosquito catch between the citric acid (CA) treatment and the dry ice (DI) treatment in University of Sydney, Camperdown; Figure S4: Comparison of mosquito catch between the citric acid (CA) treatment and the dry ice (DI) treatment in Newington Nature Reserve, Sydney Olympic Park.
