# Supplementary material for: Citric Acid and Sodium Bicarbonate as an Alternative Carbon Dioxide Source for Mosquito Surveillance
Source: Insects. 2025 Jan 16;16(1):90. doi: 10.3390/insects16010090 (PMC11765980; doi:10.3390/insects16010090)
Supplement: Supplementary file 1 [file insects-16-00090-s001.zip › insects-3390765-supplementary.pdf]

Supplementary Materials

Table S1. Mosquito trapping data for University of Sydney, Camperdown, by location, date and trapping method. Ae = *Aedes*, An = *Anopheles*, Cq = *Coquillettidia*, Cx = *Culex*, Ma = *Mansonia*, Tp = *Tripteroides*, Ve = *Verrallina*

Adult Mosquito Collection Data Sheet - CO2 Trap Comparison 2024 University of Sydney **Day 1 12 February 2024**

Species/Trap Site	ACID 1	DRY-ICE 1	ACID 2	DRY-ICE 2	ACID 3	DRY-ICE 3	ACID 4	DRY-ICE 4	TOTAL	Mean ACID	Mean DRY-ICE
Ad.venustipes									0	0	0
Ae. aculeatus									0	0	0
Ae.alboannulatus									0	0	0
Ae.alternans									0	0	0
Ae.australis									0	0	0
Ae.camptorhynchus									0	0	0
Ae.eidsvoldensis									0	0	0
Ae.flavifrons									0	0	0
Ae.imperfectus									0	0	0
Ae.mallochi									0	0	0
Ae. nr. monocellatus									0	0	0
Ae.multiplex									0	0	0
Ae.notoscriptus	23	63	64	223	37	49	0	16	475	124	351
Ae.procax									0	0	0
Ae.rubrithorax									0	0	0
Ae. theobaldi									0	0	0
Ae.vigilax									0	0	0
Ae.vittiger									0	0	0
Ae. Marks #51									0	0	0
An.annulipes									0	0	0
An.atratipes									0	0	0
Cq.linealis									0	0	0
Cq. variagata									0	0	0
Cq.xanthogaster									0	0	0
Cx.annulirostris									0	0	0
Cx.australicus									0	0	0
Cx.bitaeniorhynchus									0	0	0
Cx.halifaxii									0	0	0
Cx.molestus	1				2				3	3	0
Cx.orbostiensis									0	0	0
Cx. postspiraculosus									0	0	0
Cx.quiquefasciatus	6	12	4	27	94	2	2	2	149	106	43
Cx.sitiens									0	0	0
Cx.squamosus									0	0	0
Ma.uniformis									0	0	0
Tp. atripes									0	0	0
Ur. pygmaea									0	0	0
Ve. funerea									0	0	0
Ve. Marks 52									0	0	0
**Total**	**30**	**75**	**68**	**250**	**133**	**51**	**2**	**18**	**627**	233	394
**Species Richness**	**3**	**2**	**2**	**2**	**3**	**2**	**1**	**2**	**3**	3	2

Adult Mosquito Collection Data Sheet - CO2 Trap Comparison 2024 University of Sydney **Day 2 13 February 2024**

Species/Trap Site	ACID 1	DRY-ICE 1	ACID 2	DRY-ICE 2	ACID 3	DRY-ICE 3	ACID 4	DRY-ICE 4	TOTAL	Mean ACID	Mean DRY-ICE
Ad.venustipes									0	0	0
Ae. aculeatus									0	0	0
Ae.alboannulatus									0	0	0
Ae.alternans									0	0	0
Ae.australis									0	0	0
Ae.camptorhynchus									0	0	0
Ae.eidsvoldensis									0	0	0
Ae.flavifrons									0	0	0
Ae.imperfectus									0	0	0
Ae.mallochi									0	0	0
Ae. nr. monocellatus									0	0	0
Ae.multiplex									0	0	0
Ae.notoscriptus	52	2	23	42	23	10	4	0	156	102	54
Ae.procax									0	0	0
Ae.rubrithorax									0	0	0
Ae. theobaldi									0	0	0
Ae.vigilax									0	0	0
Ae.vittiger									0	0	0
Ae. Marks #51									0	0	0
An.annulipes									0	0	0
An.atratipes									0	0	0
Cq.linealis									0	0	0
Cq. variagata									0	0	0
Cq.xanthogaster									0	0	0
Cx.annulirostris				1					1	0	1
Cx.australicus									0	0	0
Cx.bitaeniorhynchus									0	0	0
Cx.halifaxii									0	0	0
Cx.molestus	2					1			3	2	1
Cx.orbostiensis									0	0	0
Cx. postspiraculosus									0	0	0
Cx.quiquefasciatus	69	2	15	23	19	62	17	9	216	120	96
Cx.sitiens									0	0	0
Cx.squamosus									0	0	0
Ma.uniformis									0	0	0
Tp. atripes									0	0	0
Ur. pygmaea									0	0	0
Ve. funerea									0	0	0
Ve. Marks 52									0	0	0
**Total**	**123**	**4**	**38**	**66**	**42**	**73**	**21**	**9**	**376**	224	152
**Species Richness**	**3**	**2**	**2**	**3**	**2**	**3**	**2**	**1**	**4**	3	4

Table S2. Mosquito trapping data for Newington Nature Reserve, Sydney Olympic Park, by location, date and trapping method. Ae = *Aedes*, An = *Anopheles*, Cq = *Coquillettidia*, Cx = *Culex*, Ma = *Mansonia*, Tp = *Tripteroides*, Ve = *Verrallina*

Adult Mosquito Collection Data Sheet - CO2 Trap Comparison 2024 Newington Nature Reserve **Day 1 22 February 2024**

Species/Trap Site	ACID 1	DRY-ICE 1	ACID 2	DRY-ICE 2	ACID 3	DRY-ICE 3	ACID 4	DRY-ICE 4	TOTAL	Mean ACID	Mean DRY-ICE
Ad.venustipes									0	0	0
Ae. aculeatus						1	1	1	3	1	2
Ae.alboannulatus									0	0	0
Ae.alternans	5	21	9	15	5	13	3	1	72	22	50
Ae.australis									0	0	0
Ae.camptorhynchus									0	0	0
Ae.eidsvoldensis									0	0	0
Ae.flavifrons									0	0	0
Ae.imperfectus									0	0	0
Ae.mallochi									0	0	0
Ae. nr. monocellatus									0	0	0
Ae.multiplex						1			1	0	1
Ae.notoscriptus	25	12	12	14	1	1		2	67	38	29
Ae.procax			2	3	4	4		4	17	6	11
Ae.rubrithorax									0	0	0
Ae. theobaldi									0	0	0
Ae.vigilax	58	117	131	232	82	119	91	215	1045	362	683
Ae.vittiger									0	0	0
Ae. Marks #51									0	0	0
An.annulipes	3	13	5	9	3	19	4	20	76	15	61
An.atratipes									0	0	0
Cq.linealis	6	11	8	6	8	4	8	18	69	30	39
Cq. variagata									0	0	0
Cq.xanthogaster	1							1	2	1	1
Cx.annulirostris	3	31	24	40	16	28	20	37	199	63	136
Cx.australicus									0	0	0
Cx.bitaeniorhynchus									0	0	0
Cx.halifaxii									0	0	0
Cx.molestus				2				2	4	0	4
Cx.orbostiensis		1		3		1	1	1	7	1	6
Cx. postspiraculosus									0	0	0
Cx.quiquefasciatus									0	0	0
Cx.sitiens	17	61	13	18	10	12	7	11	149	47	102
Cx.squamosus									0	0	0
Ma.uniformis	1		1			1		4	7	2	5
Tp. atripes						1			1	0	1
Ur. pygmaea									0	0	0
Ve. funerea						1			1	0	1
Ve. Marks 52									0	0	0
**Total**	**119**	**267**	**205**	**342**	**129**	**206**	**135**	**317**	**1720**	588	1132
**Species Richness**	**9**	**8**	**9**	**10**	**8**	**14**	**8**	**13**	**16**	12	16

Species/Trap Site	ACID 1	DRY-ICE 1	ACID 2	DRY-ICE 2	ACID 3	DRY-ICE 3	ACID 4	DRY-ICE 4	TOTAL	Mean ACID	Mean DRY-ICE
Ad.venustipes									0	0	0
Ae. aculeatus			1		1	2	3	1	8	5	3
Ae.alboannulatus									0	0	0
Ae.alternans	14	12	19	29	6	30	4	3	117	43	74
Ae.australis									0	0	0
Ae.camptorhynchus									0	0	0
Ae.eidsvoldensis									0	0	0
Ae.flavifrons									0	0	0
Ae.imperfectus									0	0	0
Ae.mallochi									0	0	0
Ae. nr. monocellatus									0	0	0
Ae.multiplex			1						1	1	0
Ae.notoscriptus	24	13	12	33	1	4	4	2	93	41	52
Ae.procax	2	2		4	4	2		1	15	6	9
Ae.rubrithorax									0	0	0
Ae. theobaldi									0	0	0
Ae.vigilax	197	267	83	513	205	307	183	263	2018	668	1350
Ae.vittiger									0	0	0
Ae. Marks #51									0	0	0
An.annulipes	5	16	1	72	1	13		23	131	7	124
An.atratipes									0	0	0
Cq.linealis	19	29	9	64	12	23	21	23	200	61	139
Cq. variagata									0	0	0
Cq.xanthogaster		1	1						2	1	1
Cx.annulirostris	48	28	16	69	41	28	32	27	289	137	152
Cx.australicus									0	0	0
Cx.bitaeniorhynchus									0	0	0
Cx.halifaxii									0	0	0
Cx.molestus						1			1	0	1
Cx.orbostiensis					1				1	1	0
Cx. postspiraculosus									0	0	0
Cx.quiquefasciatus		1		4				2	7	0	7
Cx.sitiens	8	20	12	17	21	19	14	13	124	55	69
Cx.squamosus									0	0	0
Ma.uniformis		1		1					2	0	2
Tp. atripes									0	0	0
Ur. pygmaea									0	0	0
Ve. funerea							1		1	1	0
Ve. Marks 52									0	0	0
**Total**	**317**	**390**	**155**	**806**	**293**	**429**	**262**	**358**	**3010**	1027	1983
**Species Richness**	**8**	**11**	**10**	**10**	**10**	**10**	**8**	**10**	**16**	13	13

Table S3. CO_2 cost per night per source, February 2024. Operational cost (1 night) excludes cargo change

Agent	Quantity sold	Unit cost	Quantity used for 1 night	Source	Operational cost (1 night)
Dry Ice	Variable	$12/kg	1.5kg	Dry Ice Australia	$18
				Industrial supply	
		$8/kg			$12
Citric Acid	10kg	$62	500g	BLANTS Wellbeing & Lifestyle	$3.1
$NaHCO_3$	10kg	$54	500g	BLANTS Wellbeing & Lifestyle	$2.7

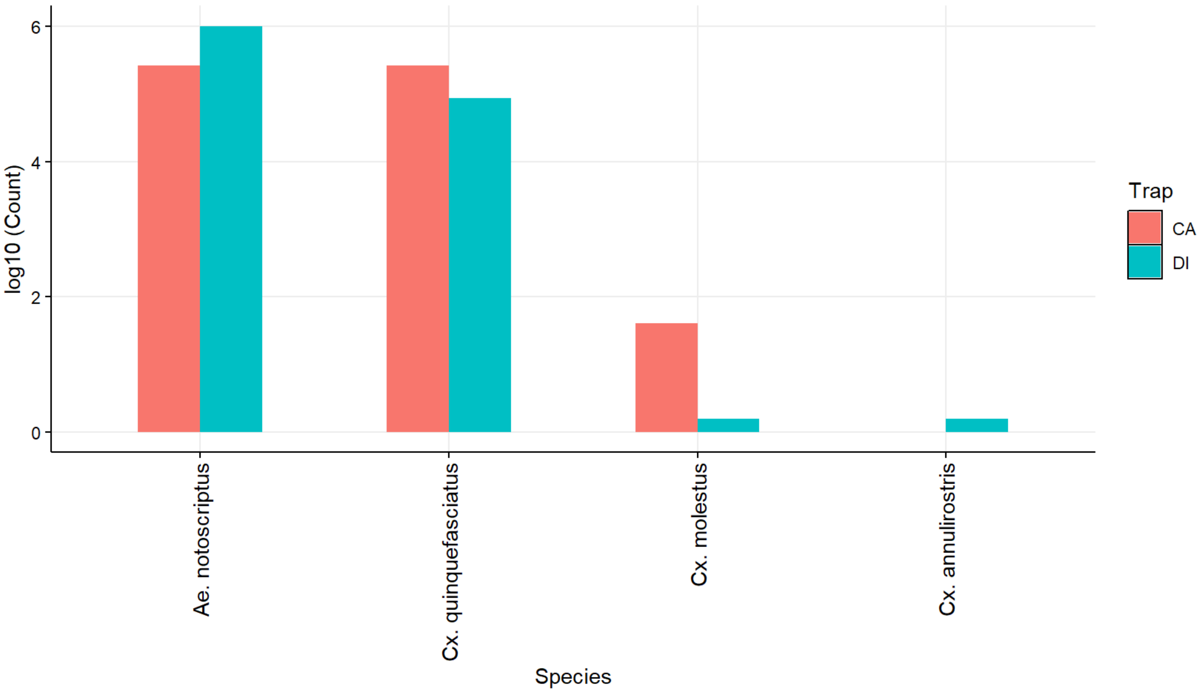

Figure S1 Distribution of mosquito species captured at the University of Sydney, Camperdown, using citric acid-based (CA) and dry ice-based (DI) traps. The bar plot illustrates the total count of four mosquito species. The y-axis mosquito count is presented in a log10 scale to facilitate visualisation of differences in species counts. Ae = *Aedes*, Cx = *Culex*

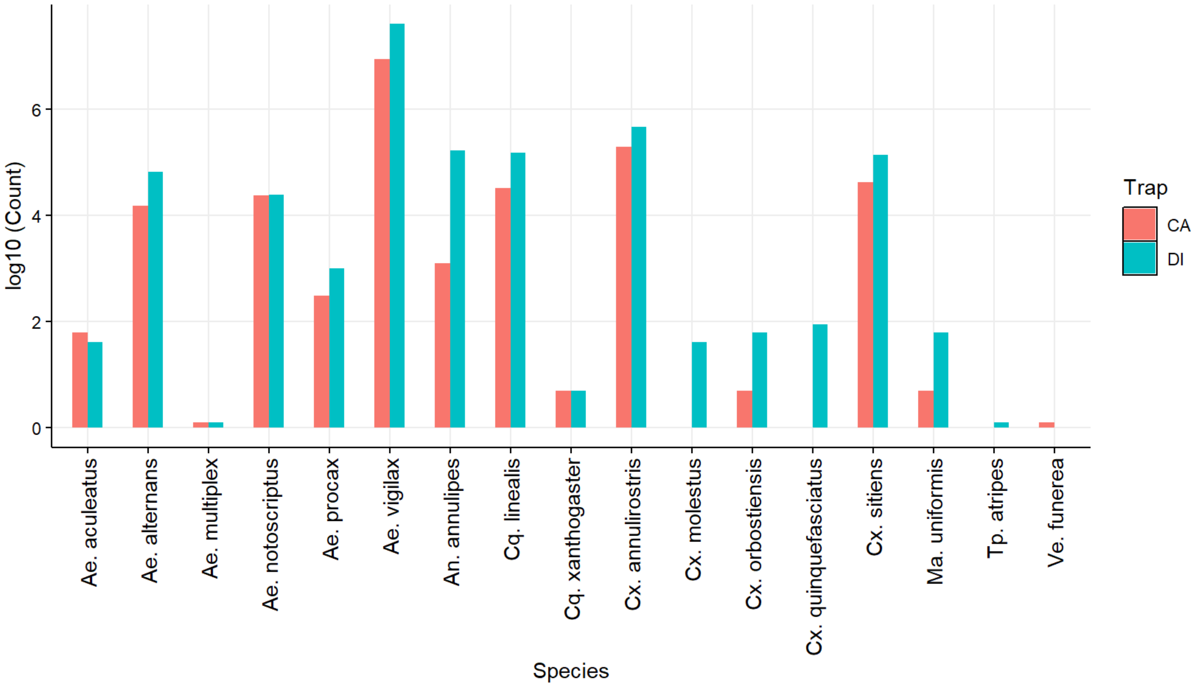

Figure S2 Distribution of mosquito species captured in Newington Nature Reserve using citric acid-based (CA) and dry ice (DI)-based traps. The bar plot illustrates the total count of seventeen mosquito species. The y-axis mosquito count is presented in log10 scale to facilitate visualisation of differences in species counts. Ae = *Aedes*, An = *Anopheles*, Cq = *Coquillettidia*, Cx = *Culex*, Ma = *Mansonia*, Tp = *Tripteroides*, Ve = *Verrallina*

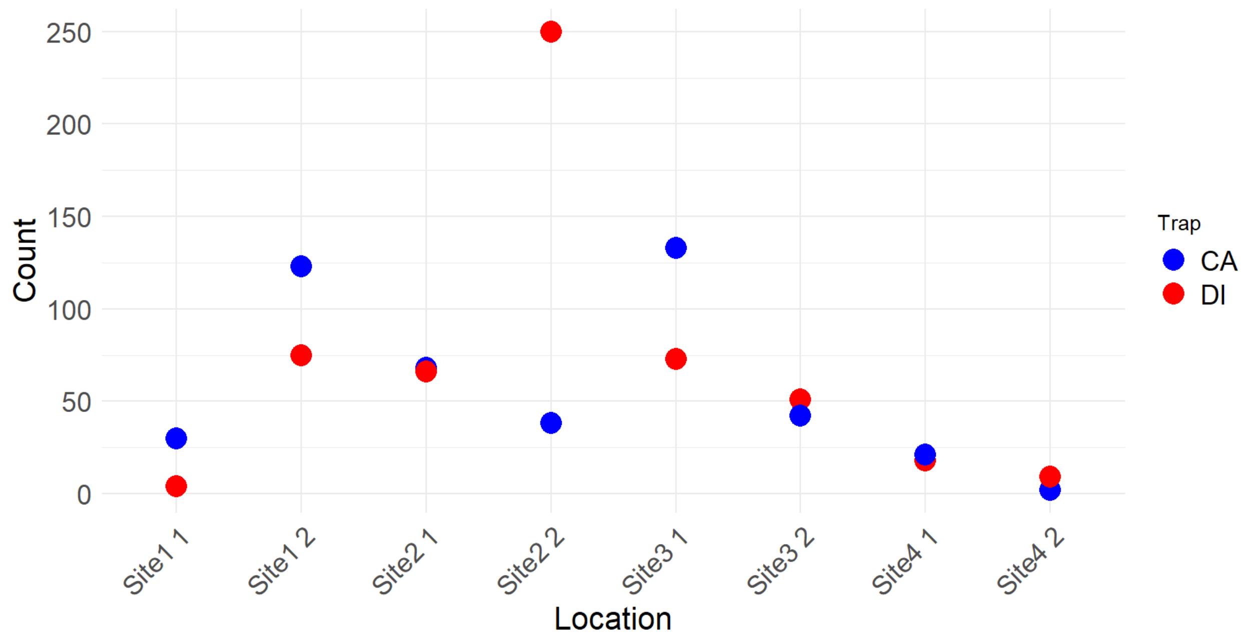

Figure S3 Comparison of mosquito catch between the citric acid (CA) treatment and the dry ice (DI) treatment in University of Sydney, Camperdown.

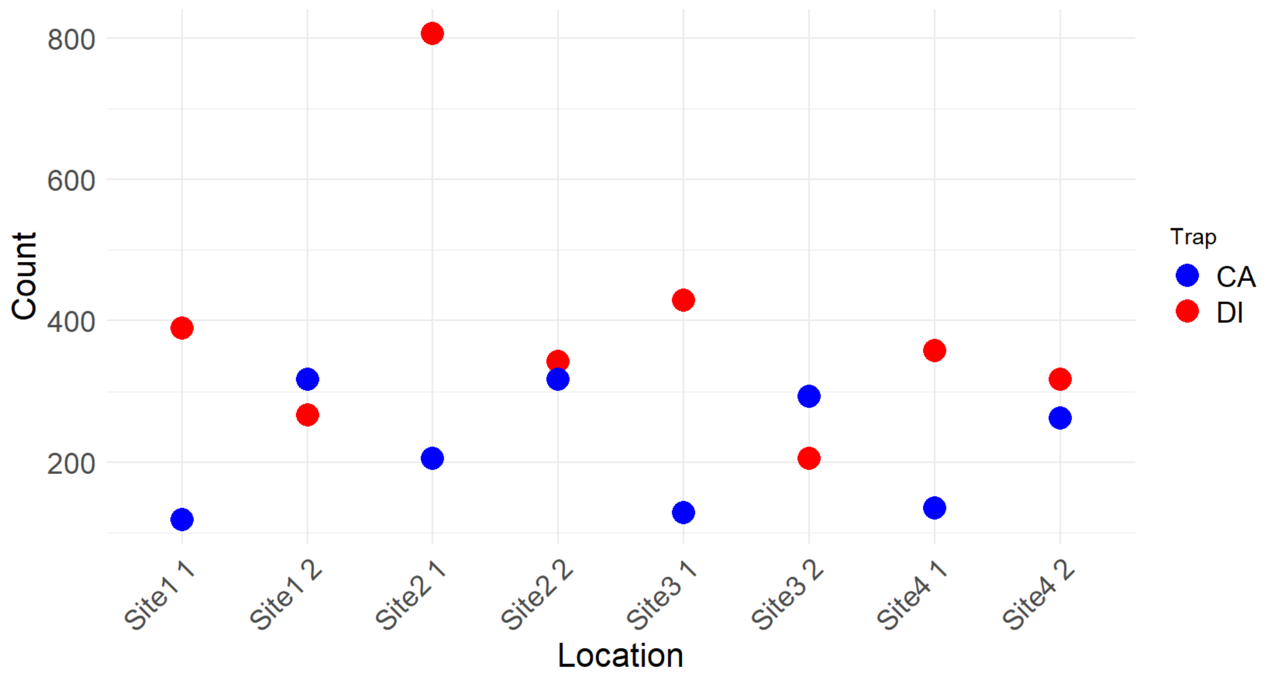

Figure S4 Comparison of mosquito catch between the citric acid (CA) treatment and the dry ice (DI) treatment in Newington Nature Reserve, Sydney Olympic Park.
